# Medical errors in primary care clinics – a cross sectional study

**DOI:** 10.1186/1471-2296-13-127

**Published:** 2012-12-26

**Authors:** Ee Ming Khoo, Wai Khew Lee, Sondi Sararaks, Azah Abdul Samad, Su May Liew, Ai Theng Cheong, Mohd Yusof Ibrahim, Sebrina HC Su, Ainul Nadziha Mohd Hanafiah, Kalsom Maskon, Rohana Ismail, Maimunah A Hamid

**Affiliations:** 1 Department of Primary Care Medicine, Faculty of Medicine, University of Malaya, Jalan Lembah Pantai, Kuala Lumpur, 50603, Malaysia; 2 Luyang Health Clinic, Ministry of Health Malaysia, Off Jalan Lintas, Kota Kinabalu, Sabah, Malaysia; 3 Department of Health Outcomes Research, Institute for Health Systems Research, Ministry of Health Malaysia, Jalan Rumah Sakit, Bangsar, Kuala Lumpur, Malaysia; 4 Pantai Health Clinic, Ministry of Health Malaysia, Wisma Goshen, Plaza Pantai, Off Jalan Pantai Bharu, Kuala Lumpur, Malaysia; 5 Department of Family Medicine, Universiti Putra Malaysia, Serdang, Malaysia; 6 State Health Department of Sabah, Ministry of Health Malaysia, 3rd Floor, Federal Building, Jalan Mat Salleh, Kota Kinabalu, Sabah, Malaysia; 7 Department of Medical Services, Medical Care Quality Section, Development Division, Ministry of Health Malaysia, Putrajaya, Malaysia; 8 Department of Public Health, Division of Family Health Development, Ministry of Health Malaysia, Putrajaya, Malaysia; 9 Office of the Deputy Director General of Health (Research & Technical Support), Ministry of Health Malaysia, Level 12, Block E7, Parcel E, Federal Government Administrative Center, Putrajaya, Malaysia

**Keywords:** Medical errors, Diagnostic errors, Medication errors, Primary health care

## Abstract

**Background:**

Patient safety is vital in patient care. There is a lack of studies on medical errors in primary care settings. The aim of the study is to determine the extent of diagnostic inaccuracies and management errors in public funded primary care clinics.

**Methods:**

This was a cross-sectional study conducted in twelve public funded primary care clinics in Malaysia. A total of 1753 medical records were randomly selected in 12 primary care clinics in 2007 and were reviewed by trained family physicians for diagnostic, management and documentation errors, potential errors causing serious harm and likelihood of preventability of such errors.

**Results:**

The majority of patient encounters (81%) were with medical assistants. Diagnostic errors were present in 3.6% (95% CI: 2.2, 5.0) of medical records and management errors in 53.2% (95% CI: 46.3, 60.2). For management errors, medication errors were present in 41.1% (95% CI: 35.8, 46.4) of records, investigation errors in 21.7% (95% CI: 16.5, 26.8) and decision making errors in 14.5% (95% CI: 10.8, 18.2). A total of 39.9% (95% CI: 33.1, 46.7) of these errors had the potential to cause serious harm. Problems of documentation including illegible handwriting were found in 98.0% (95% CI: 97.0, 99.1) of records. Nearly all errors (93.5%) detected were considered preventable.

**Conclusions:**

The occurrence of medical errors was high in primary care clinics particularly with documentation and medication errors. Nearly all were preventable. Remedial intervention addressing completeness of documentation and prescriptions are likely to yield reduction of errors.

## Background

Medical errors have been defined as an actual or a potential serious lapse in the standard of care provided to a patient, or harm caused to a patient through the performance of a health service or a healthcare professional [1]. Makeham et al. suggested that medical errors are events that are considered as a threat to patient well-being that should not happen or recur [2]. Medical errors can be categorized as administrative, communication, diagnostic, documentation, medication, surgical or procedural, and decision-making [3].

In 1999, the report “To Err is Human” by the Institute of Medicine alerted the medical fraternity to the seriousness of medical errors and the adverse events that occurred in medical practice [4]. In the US, a study showed a prevalence rate of adverse events of 3.7 per 100,000 clinic visits; 83% of which were preventable [5]. The World Health Organization (WHO) [6] has also estimated that millions of people suffer injuries directly attributable to medical care, many of which are preventable. Some countries like the United Kingdom consider recognition and reduction of potential harm as a priority for health care providers [7].

There is a lack of studies published on medical errors in primary care. Many aspects of primary care such as early presentation of undifferentiated disease [8], the varied patient population and characteristics, and the multiplicity of caregivers and sites of care render the study of medical errors to be complex [9]. This is further complicated by the different error reporting methods, definitions and classifications of types of medical errors used by researchers [2].

A review of 11 studies conducted in primary care settings found that the rates of medical errors ranged between 5 and 80 errors per 100,000 visits [7]. The most common errors were those related to delayed or missed diagnoses, followed by treatment errors.

Malaysia is geographically divided into East and West Malaysia by the South China Sea. There are 806 government primary care clinics in Malaysia with 270 in East Malaysia and 536 in West Malaysia [10]. Primary medical care services in these clinics are mainly provided by medical assistants. These are health care providers with a Diploma in Medical Assistance, a 3-year medical training programme that allows independent or limited supervision in patient care provision. A number of clinics are led by medical officers who are doctors with no specialized training; and family medicine specialists, who are trained family physicians. To date, there has been no published studies on medical errors in primary care in Malaysia. The aim of this study is to determine the prevalence and magnitude of medical errors, including diagnostic inaccuracies and management errors, in a public funded primary care setting.

## Methods

Twelve government primary care clinics were selected by purposive sampling based on geographical region and level of expertise of main health care provider. Six clinics from East Malaysia and 6 from West Malaysia were selected. In each region, 2 clinics each, headed by health care practitioners at the 3 different levels of expertise (family medicine specialists, medical officers and medical assistants) were chosen. In this cross-sectional study, medical records were selected by systematic random sampling from each clinic and photocopied in April 2007. All identifiers of patients, health care providers and clinics were deleted. Each medical record was subsequently reviewed independently by 2 family medicine specialists. Disagreements in assessments were reconciled by discussion and consensus, with arbitration from the research team members when necessary.

The operational definition of errors used in this study is as follows: Diagnostic errors were deemed to have occurred when the history or physical examination did not match the problem or diagnosis stated in the medical records. Management errors were deemed to have occurred if there was an error in investigation, medication or in the decision making process. Documentation errors were deemed to have occurred when there were missing or inadequate documentation of history, examination, diagnosis in the medical records or problems of illegibility. Diagnosis and management errors were deemed inconclusive when the reviewers could not reach a conclusion due to illegibility, insufficient information or poor documentation. The main outcomes measured were frequency and types of medical errors such as diagnostic, management and documentation errors. In addition, the potential of an error to cause serious harm and the possibility of error prevention were also assessed. The likelihood of an error to cause serious morbidity/mortality for each record was assessed based on the opinion of a team of two expert panel reviewers comprising family medicine specialists, and was a Yes or No response. The panel was asked to consider the likelihood of harm for both short and long term complications. Preventability of error was assessed similarly for each record as to whether the error that had occurred was preventable. The panel was asked to rate their degree of confidence in the evidence for preventability by using a subjective assessment based on a 6-point scale from ‘virtually no evidence for preventability’ to ‘virtually certain of evidence for preventability’.

Data was entered in EPI INFO 2000 and analyzed using STATA SE version 10. We computed variables for the different errors measured according to the definitions above, and point estimates were derived for each clinic. As the clinics differed from each other, we pooled the error estimates across all the clinics studied using meta-analysis methods in STATA SE version 10.

Ethical approval for this study was obtained from the Medical Research & Ethics Committee, Ministry of Health Malaysia (MREC MRG-07-LOI-HSR-1) and permission from the District Health Officer in charge of each participating clinic.

## Results

The retrieval rate of medical records sampled was 99.1%. The location and response or retrieval rate of medical records for each clinic is shown in Table 1. Most patient encounters (81%) were with medical assistants. Only 17.9% patient encounters were handled by medical officers and 1.1% by family medicine specialists.

**Table 1:** Location and retrieval rate of medical records of participating clinics

**Clinic code**	**Total medical records retrieved**	**Total medical records selected**	**Total response/ retrieval rate**
	**(a)**	**(b)**	= a/b *100
**Overall Response Rate**	**1753**	**1769**	**99.1**
**WM_FMS**	133	135	98.5
**WM _FMS**	156	156	100.0
**WM _MO**	151	152	99.3
**WM _MO**	111	116	95.7
**WM _MA**	141	141	100.0
**WM _MA**	102	102	100.0
**EM_FMS**	155	157	98.7
**EM _FMS**	151	156	96.8
**EM _MO**	157	157	100.0
**EM _MO**	171	171	100.0
**EM _MA**	167	167	100.0
**EM _MA**	158	159	99.4

*WM* West Malaysia, EM: East Malaysia.

*FMS* Family Medicine Specialist, *MO* Medical Officer, *MA* Medical Assistant.

Table 2 summarizes the type and frequencies of the errors.

**Table 2:** Errors detected in audited medical records

	**N**	**Error rate**		**Inconclusive rate**	
		**%**	**95% CI (%)**	**%**	**95% CI (%)**
**Overall documentation errors**	1753	98.0	97.0 – 99.1	-	-
No/Inadequate History	1753	46.5	29.7 – 63.2	-	-
No/Inadequate Physical examination	1753	51.2	36.3 – 66.1	-	-
No/Inadequate Diagnosis	1753	42.5	24.7 – 60.2	-	-
**Overall management errors**	1753	53.2	46.3 – 60.2	13.9	9.0 – 18.8
**Medication errors**	1753	41.1	35.8 – 46.4	27.9	20.3 – 35.4
Wrong dosage/frequency prescribed		48.5	41.5 – 55.6		
Inappropriate medication prescribed		47.2	39.0 – 55.4		
Drug interaction/adverse reactions		9.8	5.1 – 14.4		
**Investigation errors**	1753	21.7	16.5 – 26.8	22.3	14.8 – 29.7
No investigation done		72.6	68.1 – 77.1		
Unnecessary investigation done		8.6	4.7 – 12.6		
**Decision making errors**	1753	14.5	10.8 – 18.2	40.2	30.4 – 49.9
Inappropriate follow up		48.4	37.7 – 59.1		
Inappropriate care plan		8.1	4.0 - 12.2		
Referral not made when it should		6.3	3.1 – 9.4		
Referral for admission not done		3.6	0 - 7.7		
**Diagnostic errors**	1753	3.6	2.2 – 5.0	61.9	53.2 – 70.6

### Documentation errors

Overall, 98.0% (95% CI: 97.0, 99.1) of the medical records had some form of documentation problems. In nearly half of the medical records, there was no documentation of history, physical examination, presenting problem and /or diagnosis.

### Management errors

Management errors were found in 53.2% (95% CI: 46.3, 60.2) of records, with medication errors being the commonest (see Table 2). Common medication errors were wrong dosage or frequency of medication given (48.5%) and inappropriate medication given (47.2%), such as two drugs from the same categories of drugs being used (e.g. anti-histamines, non-steroidal anti-inflammatory drugs), or the use of non-evidenced based drugs (such as papase – a proteolytic enzyme supplement used for reducing oedema) (see Table 2).

Common investigatory errors were ‘no investigations performed’ when investigations were warranted (72.6%), or ‘unnecessary investigations performed’ (8.6%) (See Table 2).

Common decision making errors were inappropriate follow up (48.4%), where some patients were not given an appointment date for follow up or were given follow up dates which were too late or too soon; and inappropriate care plan (8.1%), such as patients not given or not advised on appropriate management or care plans (see Table 2).

### Diagnostic errors

There were 3.6% (95% CI: 2.2, 5.0) records that had diagnostic errors. However, 61.9% of the records were inconclusive for diagnostic errors.

### Preventability and likelihood of serious harm

A total of 93.5% of all errors were considered preventable and 39.9% had a possibility of causing serious harm (see Table 3).

**Table 3:** Preventability of errors and likelihood to cause harm

	**N**	**Error rate**		**Inconclusive rate**	
		**%**	**95% CI (%)**	**%**	**95% CI (%)**
**Preventability**	1753	93.5	91.3 – 95.7	1.9	0.8 – 2.9
**Likely to cause serious harm**	1753	39.9	33.1 – 46.7		

## Discussion

### Summary of main findings

The frequency of medical errors found in the medical records in the 12 clinics in Malaysia in this cross-sectional study of 1753 records was: documentation errors 98.0%; medication errors 41.1%, investigation errors 21.7%, decision making errors 14.5% and diagnostic errors 3.6%. The majority of these patient encounters were with medical assistants. Almost all medical records had some form of documentation problem. Diagnostic and management errors were found in half of the patient records. Most errors were considered to be preventable and 40% of the errors were viewed as having a potential for causing serious harm.

This is the first study conducted on medical errors in primary care settings in Malaysia. Other studies on medical errors in primary care, used incident reporting [2,11,12] and examined the different aspects of errors [5,13-15]. We found the rate of medical errors in primary care to be unacceptably high with 40% of the errors having the potential to cause serious harm.

### Documentation errors

We found an extremely high rate of documentation error. This is in contrast to a study in the US where clinician self-reported missing information per patient visit was only 13.6% [16]. In developed countries, legibility may not be an issue because of electronic records. The primary care clinics in this study were not computerized. Another contributing factor to poor documentation could be the heavy workload at the clinics, which ranged from 1 to 13 patients per personnel per hour in participating clinics.

### Medication errors

We found the medication error to be 41.1%. Other studies have reported a wide range of error rates, ranging from less than 1% to 52% per 100 incidents reported [5,7,8,11,12,17,18]. Some studies reported treatment errors, which could be either medication errors or errors in other forms of treatments, that ranged between 15% and 47% per 100 incident/errors reported [2,6,12,15,17,18]. Common medication errors found included simultaneous prescription of two antihistamines for upper respiratory tract infections (for example, one for cough and one for nasal decongestant) and prescription of antacids with non-steroidal anti-inflammatory drugs (NSAID) for perceived prevention of NSAID-induced ulcer.

### Investigation errors

The occurrence rate of investigation errors was 21.7%, which is comparable with findings from studies done in the US and Australia [2,17]. However, when one takes into account the inconclusive errors, the occurrence rate could potentially be higher. Our definition of investigation errors included investigations that should have been done but were not ordered to be carried out. Resource constraints for investigations in some clinics are a possible contributing factor.

### Decision making errors

There were 14.5% of the records that had decision making errors. However, 40.2% of the records were inconclusive for these errors. The main error noted was inappropriate period for follow-up care. Inappropriate referrals (where referrals were inappropriately given or omitted) occurred in 6.3% of the consultations. We were unable to differentiate whether this was due to lack of awareness and knowledge or judgment errors. In some studies, judgment error was reported to range from 2% in the US to 44% in Australia [11,12,17,18].

### Diagnostic errors

Although we found a low rate of diagnostic errors (3.6%), in 61.9% of the records, the diagnosis was deemed to be inconclusive due to inadequate documentation, likely causing an underestimation of errors. A number of records had only the presenting problem or diagnosis written, with no documentation of history or physical examination. The actual rate of error is likely to be much higher because of such high inconclusive rates. Other studies have found the percentage of diagnostic errors out of total potential errors to range between 1.3% and 78% [2,5,7,8,11,17,19-21]. Apart from poor documentation, diagnostic errors could be attributed to a lack of knowledge and expertise of the health care providers. Most (81%) of the patient encounters were managed by medical assistants who provided a wide scope of medical services at a level similar to nurse practitioners in primary care clinics. Their service is important especially in the medically under-served areas. It is therefore important to provide continuing medical education to this group of practitioners.

### Preventability and likelihood to cause harm

About 40% of the errors were assessed by the expert panel of family medicine specialists as having the potential to cause serious harm, either in the short or long term. This is consistent with the findings of other studies where potential harm was deemed likely to occur in 5.8% to 49% [15,17]. Most of the errors were perceived to have strong evidence for preventability, which again was consistent with findings from other studies [5,7,12,22]. In view of the high rate of potential serious harm, it is imperative for urgent measures to be taken to reduce the rate of medical errors in primary care.

Currently in Malaysia, there is lack of a formal reporting mechanism for medical errors in primary care. Incident reporting is practiced as a self-reporting process, and the number of incidents reported is influenced by a “blame-free” culture. Internationally, only 30% of incidents are said to be reported [5,11]. A workable system is needed to detect and investigate errors in primary care.

### Implications for clinical practice and future research

The occurrence of medical errors in primary care was high and consistent with international literature. This study found extremely high rates of documentation errors and this should be the area of greatest priority for intervention. Interventions targeting legibility of handwriting or structured or electronic record keeping are possible system changes that can be considered. Medication errors can be reduced by the use of a tool or drug formulary that summarizes information on commonly used drugs, their dosages and frequencies for use.

The likelihood of errors to possibly cause serious harm was high, of which most were preventable. A concerted effort is needed to improve patient safety in primary care that includes system changes, continuous education, monitoring of implementation and policy change.

The findings of this study on medical errors call for more studies of this nature as well as interventional studies to reduce medical errors. Further research is needed to identify organizational determinants and latent failures to improve the system. A review of interventions that were successful elsewhere is needed and their applicability to the local context is required. In addition, the magnitude of errors in general practice in the private sector is unknown and should be studied.

### Strengths and limitations of the study

Selection of the clinics was by purposive sampling. An equal number of clinics, supervised by different levels of expertise, were selected from two different geographical regions to increase the validity of this study. In addition, the medical records were selected randomly.

We assessed errors by reviewing medical records, and not on errors reported by practitioners. This method of error detection would give a better reflection of the extent of medical errors as opportunistic incidents reporting tend to underestimate errors [7].

We were not able to detect all adverse events due to misdiagnosis, delayed diagnosis or missed diagnosis as this was a cross-sectional study. Therefore, diagnostic errors could have been underestimated. We also did not study patient factors that could contribute to errors in management such as patient’s request for investigations, refusals for referral or for admissions, which could affect the rate of decision making error. Furthermore, the majority of patient encounters in the medical records assessed were with medical assistants, with only a small number of encounters with medical officers and family medicine specialists.

Poor documentation was a major problem in this study, with the possibility that the magnitude of errors was underestimated. This is however a very important finding which requires immediate remedial action. The medico-legal implications are of concern.

This study aimed to identify areas of errors that have occurred. We did not examine the root causes of errors from which a definitive intervention could be instituted. Further research should study the causes of errors and investigate possible corrective interventions.

## Conclusion

The occurrence of medical errors in primary care was common and the likelihood of errors causing serious harm was high, of which most were considered preventable. Attention to complete documentation as well as attention to prescriptions are actions that are likely to yield immediate reduction of errors. A concerted effort is needed to improve patient safety in primary care. This should include system changes, continuous education, monitoring of implementation and policy change. This study calls for more research into the prevalence of categories of errors, and interventional studies to reduce medical errors.

## Competing interests

The authors declare no competing interests.

## Authors’ contributions

All the authors listed in the manuscript have contributed to the design, data collection, analysis and writing of the final manuscript. KEM, LWK and SS wrote the first draft. All authors have read and approved the submission of the final manuscript.

## Pre-publication history

The pre-publication history for this paper can be accessed here:

http://www.biomedcentral.com/1471-2296/13/127/prepub
